# Supplementary material for: Drug repurposing for aging research using model organisms
Source: Aging Cell. 2017 Jun 16;16(5):1006–15. doi: 10.1111/acel.12626 (PMC5595691; doi:10.1111/acel.12626)
Supplement: Supplementary file 7 — Data S1 Zip‐Archive of all report cards. [file ACEL-16-1006-s007.zip › RC_DCF.pdf]

DCF

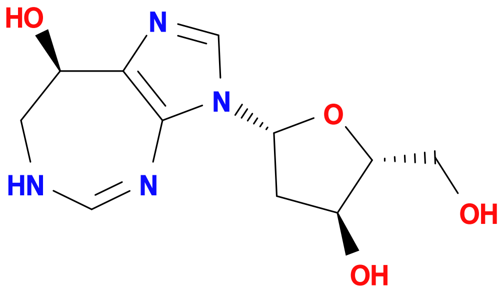

Database identifiers

ChEMBLCompound	CHEMBL1580
DrugBank	DB00552
ZINC	ZINC03806262
eMolecules	29541897

Ranking

	Rank	Score
Drosophila	NA	NA
C. elegans	283/591	0.2

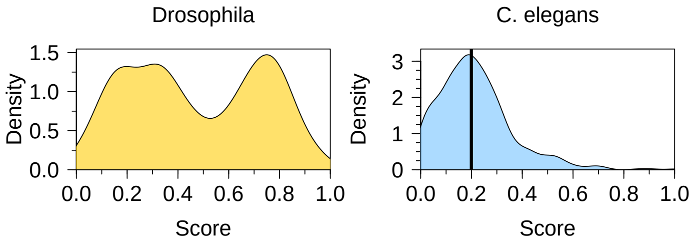

	Ageing implication	Domain conservation	Binding site conservation	Binding affinity	Bioavailability	Lipinski	Promiscuity	Purchasability	Drug approval	**Total**
Drosophila	NA	NA	NA	NA	NA	NA	NA	NA	NA	NA
C. elegans	0.792	0.853	0.965	0.0	0.21	0.0	-0.0	0.1	0.1	0.2

Names

- Nipent
- Pentostatin

Roles

ChEBI entry None has no roles Adenosine deaminase inhibitor

Status

Approved drug (according to ChEMBL)	Yes
Number of Rule of 5 violations	0
Binding affinity to original target in log units (RF-Score prediction)	nan
Burns *C. elegans* bioavailability prediction	-5.81

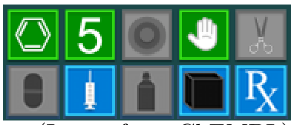

(Image from ChEMBL)

Medical Information

Indicatation: For the treatment of hairy cell leukaemia refractory to alpha interferon.
Mechanism of action: Pentostatin is a potent transition state inhibitor of adenosine deaminase (ADA), the greatest activity of which is found in cells of the lymphoid system. T-cells have higher ADA activity than B-cells, and T-cell malignancies have higher activity than B-cell malignancies. The cytotoxicity that results from prevention of catabolism of adenosine or deoxyadenosine is thought to be due to elevated intracellular levels of dATP, which can block DNA synthesis through inhibition of ribonucleotide reductase. Intracellular activation results in incorporation into DNA as a false purine base. An additional cytotoxic effect is related to its incorporation into RNA. Cytotoxicity is cell cycle phase-specific (S-phase).
Toxicity: LD50=128 mg/kg (mouse), side effects include lethargy, rash, fatigue, nausea and myelosuppression.
Metabolism: Primarily hepatic, but only small amounts are metabolized.
Retail: Bedford Labs, Ben Venue Laboratories Inc., Hospira Inc., JHP Pharmaceuticals LLC, SuperGen Inc.
Nipent 10 mg vial - 1891.31 USD / vial
Pentostatin 10 mg vial - 2280.0 USD / vial

(Information from DrugBank)

Compound Target Characteristics

Adenosine deaminase

Best gene implication in ageing for this target family came from gene Q920P6 annotated in UniProt release 2014_02. Annotation GO 7568 (aging) was Inferred from Expression Pattern

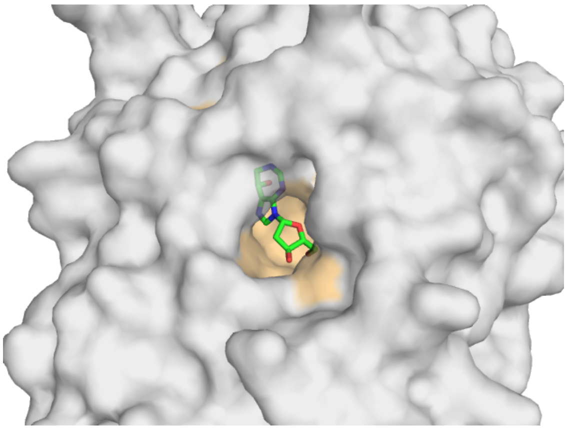

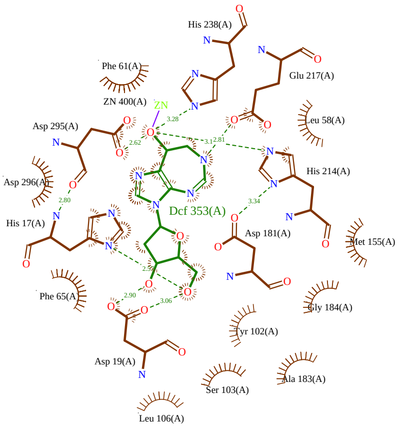

protein	amino acids contacts (binding site)																
PDB:1a4l:chainA:P03958	H	H	D	L	F	F	Y	S	L	M	A	G	H	E	H	D	D
sp:P00813:ADA_HUMAN	H	H	D	L	F	F	Y	S	L	M	A	G	H	E	H	D	D
sp:Q920P6:ADA_RAT	H	H	D	L	F	F	Y	S	L	M	A	G	H	E	H	D	D
sp:P03958:ADA_MOUSE	H	H	D	L	F	F	Y	S	L	M	A	G	H	E	H	D	D
tr:Q4FK28:Q4FK28_MOUSE	H	H	D	L	F	F	Y	S	L	M	A	G	H	E	H	D	D
tr:Q86NI2:Q86NI2_CAEEL	H	H	D	L	V	F	Y	S	L	I	A	G	H	E	H	D	D
tr:H2KYI5:H2KYI5_CAEEL	H	H	D	L	V	F	Y	S	L	I	A	G	H	E	H	D	D
sp:P53909:ADE_YEAST	H	H	E	L	F	-	Y	D	-	L	D	S	H	E	H	D	D

protein	whole protein ident	simil	domain-based ident	simil	contact-based ident	simil
PDB:1a4l:chainA:P03958	1.0	1.0	1.0	1.0	1.0	1.0
sp:P00813:ADA_HUMAN	0.81	0.93	0.83	0.96	1.0	1.0
sp:Q920P6:ADA_RAT	0.94	0.99	0.94	0.99	1.0	1.0
sp:P03958:ADA_MOUSE	1.0	1.0	1.0	1.0	1.0	1.0
tr:Q4FK28:Q4FK28_MOUSE	1.0	1.0	1.0	1.0	1.0	1.0
tr:Q86NI2:Q86NI2_CAEEL	0.39	0.76	0.4	0.78	0.88	0.97
tr:H2KYI5:H2KYI5_CAEEL	0.35	0.69	0.4	0.78	0.88	0.97
sp:P53909:ADE_YEAST	0.26	0.68	0.27	0.68	0.59	0.57
